# Biomarkers in Primary Systemic Vasculitides: Narrative Review

**DOI:** 10.3390/ijms27020730

**Published:** 2026-01-11

**Authors:** Mario Sestan, Martina Held, Marija Jelusic

**Affiliations:** 1 Department of Pediatrics, University of Zagreb School of Medicine, University Hospital Centre Zagreb, 10000 Zagreb, Croatia; mario.sestan@gmail.com; 2 Department of Internal Medicine, University of Zagreb School of Medicine, University Hospital Centre Zagreb, 10000 Zagreb, Croatia; martinaheld17129@gmail.com

**Keywords:** vasculitis, biomarkers, large-vessel vasculitis, ANCA-associated vasculitis, Kawasaki disease, IgA vasculitis, pentraxin-3, matrix metalloproteinase-9, S100 proteins, endothelial injury

## Abstract

Vasculitides are a heterogeneous group of disorders characterized by inflammation of blood vessel walls, leading to tissue ischemia and organ injury. Traditional inflammatory markers such as the erythrocyte sedimentation rate (ESR) and C-reactive protein (CRP) are widely used but lack diagnostic specificity. This has driven the search for more informative biomarkers across vasculitis subtypes. This review summarizes current evidence for validated and emerging biomarkers in large-, medium-, small-, and variable-vessel vasculitis, as well as single-organ vasculitis. Key analytes reflect systemic inflammation, such as serum amyloid A (SAA) and interleukin-6 (IL-6), as well as endothelial activation, complement pathways, neutrophil and macrophage activation, and organ-specific damage. Promising candidates include pentraxin-3 (PTX3) and matrix metalloproteinase-9 (MMP-9) in large-vessel vasculitis; N-terminal pro-B-type natriuretic peptide (NT-proBNP) and S100 proteins in Kawasaki disease; galactose-deficient immunoglobulin A1 (Gd-IgA1) and urinary angiotensinogen (AGT) in IgA vasculitis; and tissue inhibitor of metalloproteinases-1 (TIMP-1), S100 proteins, complement C3, and PTX3 in antineutrophil cytoplasmic antibody (ANCA)-associated vasculitis. Although these biomarkers provide mechanistic insight, most lack disease-specificity, external validation, or standardized assays. Future progress will require multicenter studies, harmonized testing, and integrated biomarker panels combined with imaging modalities to improve diagnosis, activity assessment, and monitoring.

## 1. Introduction

**Figure 1:**
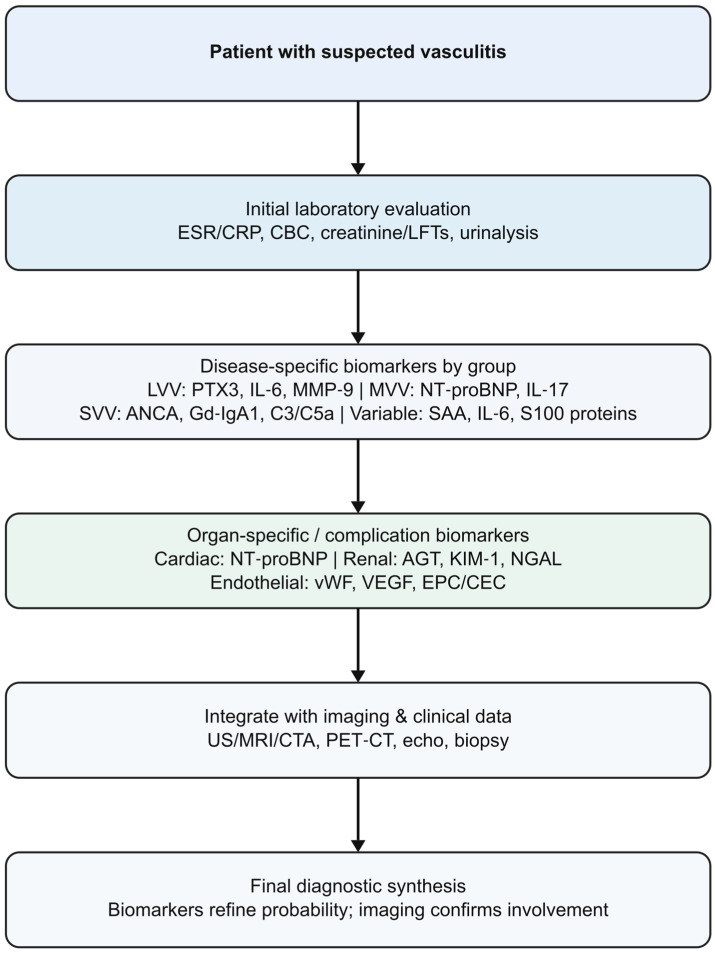
Diagnostic role of biomarkers in vasculitis. List of abbreviations: AGT: angiotensinogen; ANCA: antineutrophil cytoplasmic antibodies; C3: complement component 3; C5a: complement component C5a; CBC: complete blood count; CEC: circulating endothelial cells; CRP: C-reactive protein; CTA: computed tomography angiography; EPC: endothelial progenitor cells; ESR: erythrocyte sedimentation rate; Gd-IgA1: galactose-deficient immunoglobulin A1; IL-6: interleukin-6; IL-17: interleukin-17; KIM-1: kidney injury molecule-1; LFTs: liver function tests; LVV: large-vessel vasculitis; MMP-9: Matrix metalloproteinase-9; MRI: magnetic resonance imaging; NGAL: neutrophil gelatinase-associated lipocalin; NT-proBNP: N-terminal pro–B-type natriuretic peptide; PTX3: pentraxin-3; SVV: small-vessel vasculitis; US: ultrasound; VEGF: vascular endothelial growth factor; vWF: von Willebrand factor.

Vasculitides represent a heterogeneous group of disorders characterized by inflammation of blood vessel walls, leading to tissue ischemia and organ damage. They are classified according to the size of predominantly affected vessels as small-, medium-, or large-vessel vasculitis, with additional categories for variable-vessel vasculitis, single-organ vasculitis, vasculitis associated with systemic diseases, and vasculitis associated with probable etiology [1,2]. Although the etiology and pathogenesis differ among entities, early diagnosis remains a challenge because clinical presentations are often nonspecific and conventional laboratory markers such as ESR and CRP lack disease specificity (Figure 1). The increasing availability of advanced serologic, genetic, imaging and omics-based technologies has accelerated biomarker research in vasculitis, yet the translation of candidate biomarkers into routine practice remains limited [3,4,5,6,7,8].

**Figure 2:**
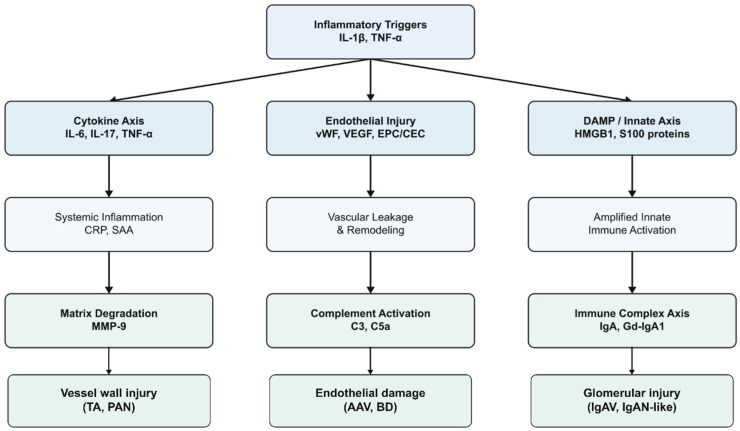
Pathogenic pathways and biomarker origins. List of abbreviations: AAV: ANCA-associated vasculitis; BD: Behçet’s disease; C3: complement component 3; C5a: complement component C5a; CEC: circulating endothelial cells; CRP: C-reactive protein; DAMP: damage-associated molecular pattern; EPC: endothelial progenitor cells; Gd-IgA1: galactose-deficient immunoglobulin A1; HMGB1: high-mobility group box-1 protein; IgA: immunoglobulin A; IgAN: IgA nephropathy; IgAV: IgA vasculitis; IL-1β: inteleukin-1β; IL-6: interleukin-6; IL-17: interleukin-17; MMP-9: Matrix metalloproteinase-9; PAN: polyarteritis nodosa; S100 proteins: S100 calcium-binding proteins; SAA: serum amyloid A; TA: Takayasu arteritis; TNF-α: tumor necrosis factor-alpha; VEGF: vascular endothelial growth factor; vWF: von Willebrand factor.

**Figure 3:**
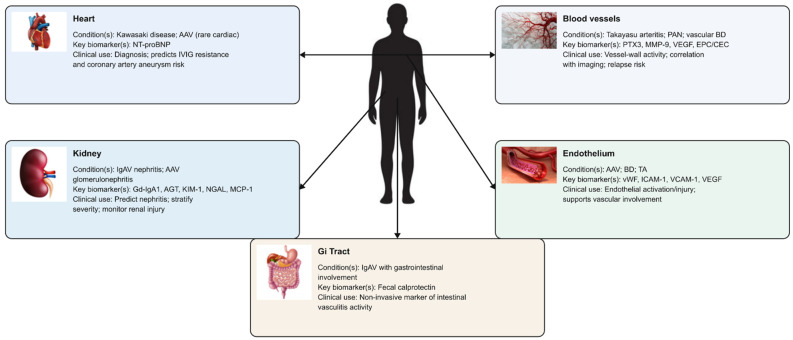
Organ-specific damage biomarkers in vasculitis. List of abbreviations: AAV: antineutrophil cytoplasmic antibody-associated vasculitis; AGT: angiotensinogen; BD: Behçet’s disease; CEC: circulating endothelial cells; EPC: endothelial progenitor cells; Gd-IgA1: galactose-deficient immunoglobulin A1; Gi: gastrointestinal; IgAV: IgA vasculitis; ICAM-1: intercellular adhesion molecule-1; IVIG: intravenous immunoglobulin; KIM-1: kidney injury molecule-1; MCP-1: monocyte chemoattractant protein-1; MMP-9: Matrix metalloproteinase-9; NGAL: neutrophil gelatinase-associated lipocalin; NT-proBNP: N-terminal pro–B-type natriuretic peptide; PAN: polyarteritis nodosa; PTX3: pentraxin-3; TA: Takayasu arteritis; VCAM-1: vascular cell adhesion molecule-1; VEGF: vascular endothelial growth factor; vWF: von Willebrand factor.

An ideal biomarker in vasculitis would improve diagnostic accuracy, differentiate disease subsets, predict disease activity or relapse, monitor treatment response, and forecast long-term outcomes. Numerous molecules reflecting inflammation, endothelial injury, immune activation, complement activation, tissue damage and genetic or epigenetic alterations have been proposed (Figure 2). However, only a few biomarkers have consistently demonstrated useful performance across cohorts, and even these require careful interpretation in the clinical context (Figure 3).

Recent years have seen a growing number of narrative and time-restricted reviews summarizing advances in systemic vasculitis, including annual overviews and thematic syntheses addressing disease classification, pathogenesis, and therapeutic developments [2,3,4,6,7,8]. In parallel with these efforts, increasing attention has been directed toward the identification of biomarkers that capture vascular inflammation, immune activation, endothelial injury, and organ-specific damage across vasculitis subtypes. The present review focuses on this biomarker-oriented perspective, integrating data from large-, medium-, small-, variable-vessel, and single-organ vasculitides, and highlighting both validated and emerging candidates in relation to their biological pathways and potential clinical utility, emphasizing their limitations and highlighting areas requiring further investigation (Table 1 and Table 2).

This narrative review is based on a focused and iterative appraisal of the published literature on biomarkers in primary systemic vasculitides. Relevant publications were identified through targeted searches of major biomedical databases, including PubMed, Web of Science, and Scopus, complemented by manual screening of reference lists from key reviews and original articles. Emphasis was placed on studies providing mechanistic insight, clinical relevance, or translational potential of biomarkers across different vasculitis subtypes. Rather than applying rigid inclusion or exclusion criteria, the literature was selected to reflect seminal work, recent advances, and clinically informative biomarker candidates, in line with the objectives of a narrative review.

## 2. Biomarkers in Large-Vessel Vasculitis

Large-vessel vasculitis (LVV) includes giant cell arteritis (GCA) and Takayasu arteritis (TA), two granulomatous vasculitides affecting the aorta and its major branches [1]. While sharing histopathologic similarities, they differ in age distribution, epidemiology, and vascular territories. GCA is the most common primary systemic vasculitis in adults, occurring almost exclusively after age 50, with the highest prevalence in Northern European descent [2,9]. TA predominantly affects young women under 40 years, with the highest incidence in Asian populations [2,10]. Both diseases may present with systemic inflammation and vascular complications, including stenosis, occlusion, aneurysm formation, and ischemic events involving the eyes, brain, and other organs.

### 2.1. Current Clinical Biomarkers

In GCA, erythrocyte sedimentation rate (ESR) and C-reactive protein (CRP) remain the most widely used biomarkers of systemic inflammation. Elevated ESR (>50 mm/h) is included in the 1990 American College of Rheumatology classification criteria [11]. In a cohort of biopsy-proven GCA, CRP showed slightly higher sensitivity than ESR (86.4% vs. 84.2%), while the specificity of either marker alone remained low (approximately 30%) and increased only modestly when used together (41%) [12]. Approximately 10% of biopsy-proven GCA cases present with normal ESR and CRP, illustrating their limited sensitivity [12].

Serum amyloid A (SAA), another hepatic acute phase protein induced by interleukin (IL)-1, IL-6, and tumor necrosis factor-alpha (TNF-α), is markedly elevated in untreated GCA, up to 80-fold higher than in controls, and correlates strongly with IL-6 levels [13]. Higher baseline ESR, CRP, SAA, and other acute-phase reactants have been linked to increased relapse risk [14].

In TA, ESR and CRP are similarly used to assess systemic inflammation. Between 10 and 30% of patients with clinically active TA have normal levels of these markers [15]. In TA patients with coronary artery involvement, baseline high-sensitivity CRP independently predicted major cardiovascular events [16]. Elevated ESR and CRP have also been associated with prolonged time to remission and higher relapse risk [17]. SAA in TA has shown higher concentrations in active disease compared with inactive disease and healthy controls, decreasing with treatment, although overlap limits accuracy [18].

Complete blood count-derived indices such as CRP/albumin ratio, neutrophil–lymphocyte ratio (NLR), platelet–lymphocyte ratio (PLR), monocyte–lymphocyte ratio (MLR), and red cell distribution width (RDW) are increased in TA compared with controls and correlate with CRP and ESR [19]. These indices reflect systemic inflammation but lack specificity.

### 2.2. Emerging Biomarkers

Pentraxin-3 (PTX3) is produced locally by endothelial cells, macrophages, and dendritic cells and stored in neutrophil granules. In TA, PTX3 levels are elevated compared with controls and outperform CRP and ESR in distinguishing active from inactive disease in some cohorts [20,21]. PTX3 also correlates with vascular enhancement and angiographic progression, reflecting local vessel-wall inflammation rather than hepatic acute-phase responses [22].

Matrix metalloproteinase-9 (MMP-9), a mediator of extracellular matrix degradation, is significantly increased in active TA and decreases with treatment, correlating with disease activity and inflammatory markers [23]. Together, PTX3 and MMP-9 provide insight into vessel-wall injury and may be less susceptible to glucocorticoid suppression.

Elevated IL-6, TNF-α, IL-18, and other cytokines are consistently reported in active TA, though correlations with clinical or imaging activity vary [17,24,25,26,27]. The T helper 17 cells (Th17)/IL-23 axis is particularly relevant; active TA demonstrates increased IL-17 and IL-23, and Th17 cell expansion tracks with disease activity [28].

Among chemokines, monocyte chemoattractant protein-1 (MCP-1) is regularly elevated in TA and correlates with disease activity scores and inflammatory markers such as ESR, CRP, and IL-6 [29].

Endothelial injury and repair biomarkers such as vascular endothelial growth factor (VEGF), endothelial progenitor cells (EPCs), and circulating endothelial cells (CECs) are increased in TA and correlate with disease activity in several studies [30,31].

Adipokines such as leptin have been associated with active TA in some cohorts, whereas adiponectin findings are inconsistent [32].

### 2.3. Clinical Utility and Limitations

ESR, CRP, and SAA remain the primary laboratory tools for assessing disease activity in LVV; however, their clinical utility is limited by poor specificity and suboptimal sensitivity, with normal values occurring in up to 10% of GCA and 10–30% of TA patients despite active disease [12,15], and by their rapid suppression following glucocorticoid therapy. Among emerging biomarkers, PTX3 shows the greatest promise because of its vascular-wall origin and its correlations with ischemic complications, imaging progression, and disease activity, while being less susceptible to glucocorticoid suppression [20,21,22]. MMP-9 provides complementary information about extracellular matrix remodeling and arterial injury [23], though its use remains constrained by assay variability and lack of standardization.

Cytokines such as IL-6 and those within the Th17/IL-17/IL-23 axis correlate with activity in both GCA and TA [24,25,26,27,28], but their measurement is limited by technical complexity and cost. S100 proteins (S100A8/A9 and S100A12) and myeloid-related protein (MRP8/14) may help differentiate active from inactive disease, especially when combined with ESR or CRP [33,34], although further validation is necessary. Endothelial biomarkers, including VEGF, ECPs, and CECs, have demonstrated associations with disease activity but inconsistent performance across studies [30,31]. Adipokines continue to be exploratory markers without confirmed diagnostic or prognostic utility [32].

Taken together, biomarker research in LVV underscores the limitations of traditional acute-phase reactants and highlights PTX3 and MMP-9 as the most promising emerging tools for assessing vascular-wall inflammation and matrix remodeling. Cytokines, chemokines, and endothelial injury markers contribute to mechanistic insight but are not ready for routine clinical use. Progress will depend on multicenter studies, harmonized assay protocols, and incorporation of biomarker data alongside imaging to improve diagnostic and monitoring strategies.

## 3. Biomarkers in Medium-Vessel Vasculitis

Medium-sized vessel vasculitides (MVV) primarily affect muscular arteries and include Kawasaki disease (KD) and polyarteritis nodosa (PAN), which differ in epidemiology, pathogenesis, and clinical presentation [1,9]. KD is an acute pediatric vasculitis predominantly affecting the coronary arteries and remains the leading cause of acquired heart disease in children worldwide [35]. PAN is a necrotizing arteritis of medium and small arteries characterized by aneurysm formation and multisystem ischemia [1]. Although numerous biomarkers have been investigated, reliable tools that enable early diagnosis, predict disease course, and stratify vascular risk remain limited.

### 3.1. Current Clinical Biomarkers in KD

Conventional inflammatory markers are widely used but lack specificity. CRP and ESR are typically elevated in acute KD, with CRP increasing early and ESR remaining high into the subacute phase, especially after IVIG administration [36]. Additional abnormalities include leukocytosis, anemia of inflammation, thrombocytosis, hypoalbuminemia, and hyponatremia.

Serum albumin is a reproducible prognostic marker. Hypoalbuminemia reflects systemic inflammation and vascular permeability and is consistently associated with IVIG resistance and coronary artery abnormalities (CAA) across multiple cohorts [35]. Complete blood count-derived indices, such as the NLR, have also shown predictive value for intravenous immunoglobulins (IVIG) resistance and early CAA formation.

NT-proBNP is the most extensively validated organ-specific biomarker in KD. Levels are significantly higher in KD than in febrile controls, including incomplete presentations, and correlate with myocardial inflammation, early coronary changes, and likelihood of IVIG non-response [37]. Despite variability in cutoff values, NT-proBNP consistently outperforms standard inflammatory markers in diagnostic and prognostic assessment.

### 3.2. Emerging Biomarkers in KD

Acute KD is characterized by increased concentrations of cytokines and other immune mediators, including IL-6, TNF-α, IL-1β, IL-10, and IL-17A, which together reflect activation of both innate and adaptive immunity [38,39]. IL-1β shows a strong association with the development of coronary arteritis and correlates with clinical severity [38], while IL-17A and the broader Th17 axis are significantly upregulated compared with febrile controls and link systemic inflammation to the risk of coronary artery abnormalities [39,40]. Soluble IL-2 receptor, a marker of T-cell activation, is also elevated in acute KD and decreases after intravenous immunoglobulin treatment, although it adds limited diagnostic value [41].

Endothelial perturbation is a prominent feature of KD, as demonstrated by elevated levels of vascular endothelial growth factor and soluble adhesion molecules such as vascular cell adhesion molecule-1 (VCAM-1), intercellular adhesion molecule-1 (ICAM-1), and E-selectin, which reflect increased vascular permeability and endothelial activation [42,43]. Circulating endothelial cells rise during acute illness and fall after treatment, indicating direct injury to the vascular endothelium [44].

Matrix-remodeling pathways contribute to coronary artery changes, with MMP-9 consistently elevated in acute KD and correlating with coronary dilation and aneurysm formation [45].

Additional biomarkers of innate immune activation include high-mobility group box 1 protein (HMGB1) and S100 proteins such as S100A8/A9 and S100A12, which are markedly increased during acute disease. S100A8/A9 helps distinguish KD from other febrile illnesses and may identify patients at risk of resistance to IVIG [46], while S100A12 reflects neutrophil-mediated vascular inflammation and is associated with early coronary involvement [47].

Fibrotic and vascular-remodeling pathways are reflected by increased galectin-3 concentrations, which correlate with the development of CAA [48]. Ferritin provides information on macrophage activation and helps identify a subset of children with macrophage activation syndrome, a hyperinflammatory phenotype associated with treatment resistance and higher risk of adverse outcomes [49].

### 3.3. Clinical Utility and Limitations of Biomarkers in KD

KD demonstrates a broad biomarker profile reflecting activation of innate and adaptive immunity, endothelial injury, extracellular-matrix degradation, and fibrotic remodelling. Despite this, no single biomarker independently confirms the diagnosis or reliably predicts coronary outcomes, and most markers are limited by nonspecificity, assay variability, or insufficient validation. CRP and ESR remain useful general inflammatory markers but are influenced by intravenous immunoglobulin treatment. Albumin and the NLR offer accessible prognostic information, while galectin-3 and ferritin highlight more severe inflammatory phenotypes but are not routinely applied.

NT-proBNP remains the most clinically useful biomarker in KD due to its ability to aid the diagnosis of incomplete KD and predict intravenous immunoglobulin resistance and early coronary artery changes. Emerging biomarkers, including IL-1β, IL-17A, VEGF, CECs, MMP-9, S100 proteins, and galectin-3, provide important mechanistic insights but require methodological standardization and multicenter evaluation before they can be incorporated into routine practice. Biomarkers, therefore, support but do not replace clinical evaluation and imaging in KD management.

### 3.4. Current Clinical Biomarkers in PAN

In PAN, ESR and CRP are frequently elevated during active disease but lack specificity and do not reliably distinguish PAN from other vasculitides [50]. Additional abnormalities—leukocytosis, anemia of inflammation, renal dysfunction, and proteinuria—reflect systemic inflammation or organ damage but do not provide disease-specific information.

### 3.5. Emerging Biomarkers in PAN

Serum HMGB1 levels are significantly elevated in PAN and may exceed those seen in other vasculitides [51]. HMGB1 correlates with CRP and markers of renal involvement.

Anti-phosphatidylserine–prothrombin antibodies (anti-PSPT) are elevated in PAN and decline with treatment [52]. Anti-moesin antibodies correlate with disease activity scores and vascular damage indices [52].

Serum lysosome-associated membrane protein 2 (LAMP-2) concentrations distinguish PAN from antineutrophil cytoplasmic antibodies (ANCA)-associated vasculitis at proposed cutoff values and correlate with disease activity [53]. Anti-LAMP-2 antibodies are also increased and show diagnostic potential [54].

Endothelial injury biomarkers, such as soluble VCAM-1, are elevated in small PAN cohorts and reflect endothelial injury or activation [55]. Anti-endothelial cell antibodies (AECA) support an immune-mediated mechanism but lack specificity.

Matrix metalloproteinases, such as MMP-3, may contribute to arterial wall destruction in PAN, though direct clinical evidence is limited [56].

### 3.6. Clinical Utility and Limitations of Biomarkers in PAN

No available biomarker reliably establishes the diagnosis of PAN or consistently distinguishes it from mimics such as deficiency of adenosine deaminase 2 (DADA2) or hepatitis-associated arteritis. Traditional markers such as ESR and CRP reflect systemic inflammation but lack specificity. HMGB1 may help differentiate PAN from other vasculitides and correlates with markers of renal involvement, though studies remain small. Anti-phosphatidylserine–prothrombin and anti-moesin antibodies correlate with disease activity and vascular damage but require larger validation.

LAMP-2 and anti-LAMP-2 antibodies show the greatest promise for diagnostic use, as proposed concentrations may distinguish PAN from ANCA-associated vasculitis, though methodological variability limits their application. Endothelial injury markers such as soluble VCAM-1, thrombomodulin, and anti-endothelial cell antibodies offer mechanistic insight but insufficient diagnostic discrimination. Overall, PAN currently lacks validated disease-specific biomarkers, and multicenter studies are needed to clarify which emerging candidates may ultimately support diagnosis or prognosis.

## 4. Biomarkers in Small-Vessel Vasculitis

Small-vessel vasculitides include ANCA-associated vasculitis (AAV) and immune complex–mediated diseases such as IgA vasculitis (IgAV), cryoglobulinemic vasculitis, and hypocomplementemic urticarial vasculitis [1]. IgAV is the most common systemic vasculitis in childhood, although it also affects adults, and is characterized by IgA-containing immune complex deposition with predominant skin, gastrointestinal, and renal involvement [57,58]. Biomarker research is particularly active in IgAV, especially regarding early prediction of nephritis.

### 4.1. Current Clinical Biomarkers in IgAV

Conventional inflammatory markers such as leukocyte count, CRP, IL-6, and SAA are elevated in children with IgAV compared with controls, and composite biomarker indices combining SAA, IgA, IgM, and CRP have shown diagnostic potential [59]. Adults similarly demonstrate increased IL-6 and SAA levels [60]. CRP, however, reflects nonspecific inflammation and does not reliably predict organ involvement [61].

Pro-inflammatory cytokines, including IL-17A, IL-18, and IL-23, are elevated in the acute phase of IgAV, supporting a role for Th17-skewed immunity [62,63]. TNF-α is increased in both pediatric and adult IgAV and correlates with renal impairment and disease severity [64,65]. Serum calprotectin (S100A8/A9) correlates with disease activity indices such as the Paediatric Vasculitis Activity Score (PVAS) and may offer greater specificity for vasculitic inflammation than CRP [66].

HMGB1 is also elevated in IgAV and is strongly expressed in lesional endothelium, inducing IL-6 and TNF-α release [67]. Higher serum HMGB1 levels are associated with nephritis in both children and adults and correlate with inflammatory and coagulation markers [68,69]. Urinary HMGB1 remains elevated in children with IgAV nephritis (IgAVN) during follow-up and correlates with hematuria and albuminuria [70].

### 4.2. Emerging Serum Biomarkers for Nephritis (IgAVN)

Galactose-deficient IgA1 (Gd-IgA1) is the most consistently validated serum biomarker for IgAVN. Pediatric and adult cohorts demonstrate higher Gd-IgA1 levels in patients with nephritis than in those without renal involvement, reflecting pathogenic overlap with IgA nephropathy [71,72,73,74,75,76,77]. A recent mechanistic study further confirmed interactions between Gd-IgA1, HMGB1, receptor for advanced glycation end-products (RAGE), and epithelial barrier molecules in IgAV [70].

Other serum markers linked to nephritis include PTX3, which predicts early IgAVN in children [78], and α-smooth muscle actin and c-Met, which correlate with histologic severity and may reflect mesangial and tubular injury [79]. Lower apolipoprotein M levels have been described in pediatric IgAV and IgAVN, suggesting impaired endothelial protective pathways [80]. Proteomic studies identified elevated serum angiotensinogen (AGT) as a marker of IgAVN, possibly reflecting activation of the intrarenal renin–angiotensin system [81].

In adult IgAVN, TNF-α levels associate with renal function impairment and biopsy severity [82].

### 4.3. Emerging Urinary and Renal Biomarkers in IgAV

Urinary biomarkers show strong potential for early identification of nephritis. Complement components, including urinary C3, C4, C5, and C5a, are increased in children with IgAVN, indicating complement activation within the kidney [83]. A meta-analysis found serum and urinary MMP-9 levels associated with nephritis risk [84].

Urinary AGT is one of the most informative markers. Children with IgAVN have significantly higher urinary AGT at onset, with greater levels in those presenting with proteinuria; values correlate with proteinuria and serum creatinine and often remain elevated during convalescence [85]. Urinary macrophage migration inhibitory factor (MIF) is similarly increased and correlates with microalbuminuria [86].

A systematic review identified kidney injury molecule-1 (KIM-1), MCP-1, N-acetyl-β-D-glucosaminidase (NAG), and AGT as the most promising urinary biomarkers in pediatric IgAVN [87]. Erythrocyte glutathione S-transferase (e-GST) is increased in children with IgAVN at onset and remains elevated for months, suggesting persistent tubular dysfunction [88].

In adult IgAVN, urinary IL-1β, IL-6, IL-8, and neutrophil gelatinase-associated lipocalin (NGAL) distinguish renal involvement from isolated extrarenal disease, and urinary IgA is associated with poor renal outcomes [77].

### 4.4. Biomarkers of Gastrointestinal Involvement in IgAV

Markers predicting gastrointestinal (GI) involvement include increased platelet and neutrophil counts, higher NLR and PLR, and lower lymphocyte count, mean platelet volume (MPV), and MPV-to-platelet ratio (MPR) in children with significant GI disease [89,90,91,92]. Elevated NLR predicts GI bleeding in adults as well [60]. Serum procalcitonin correlates with GI bleeding risk in pediatric IgAV [61].

Fecal calprotectin is the most validated biomarker of GI involvement. Levels are markedly elevated in children with intestinal symptoms and outperform CRP and blood counts for detecting mucosal inflammation [93,94]. GI involvement is itself associated with increased nephritis risk [58].

### 4.5. Clinical Utility and Limitations of Biomarkers in IgAV

IgAV is associated with a wide range of biomarkers reflecting systemic inflammation, immune dysregulation, complement activation, endothelial injury, and renal tubular damage. Although commonly used, conventional inflammatory markers do not reliably predict organ involvement. Gd-IgA1 is the most consistently validated serum biomarker for identifying patients at higher risk of nephritis, yet standardized cutoffs and broadly available assays are still lacking.

Urinary biomarkers, such as ATG, KIM-1, MCP-1, NAG, NGAL, complement fragments, and e-GST, show strong potential for early detection and monitoring of renal involvement, although most studies remain limited by small cohorts. Damage-associated molecular patterns, including HMGB1 and S100A8/A9, correlate with systemic inflammation and renal involvement. Fecal calprotectin is the most practical and validated biomarker for detecting gastrointestinal disease.

Although promising, most biomarkers require greater standardization and multicenter validation before routine clinical use.

### 4.6. Current Clinical Biomarkers in AAV

ESR, CRP, neutrophil counts, and ANCA serology remain the most widely used biomarkers in AAV. CRP and ESR increase during active disease but lack specificity. Anti-myeloperoxidase (anti-MPO) and anti-proteinase 3 (anti-PR3) titers are essential for diagnosis and classification; rising ANCA levels, particularly reappearance of MPO-ANCA, may precede relapse in a subset of patients [95], although the results are not unequivocal [96].

Ratios integrating inflammation and nutrition have been explored: the CRP-to-albumin ratio (CAR) is an independent predictor of all-cause mortality in AAV, comparable in prognostic value to major clinical risk factors [97].

Complement activation has emerged as a key pathogenic pathway. Lower serum C3 at diagnosis is associated with poor renal prognosis, higher chronicity indices, and reduced treatment responsiveness [98]. In pediatric AAV, elevated von Willebrand factor (vWF) antigen correlates with active disease and endothelial activation [99].

Delta neutrophil index (DNI), representing immature granulocyte proportion, correlates with disease activity (BVAS) and predicts relapse risk, especially in granulomatosis with polyangiitis (GPA) and microscopic polyangiitis (MPA) [100].

### 4.7. Emerging Serum Biomarkers in AAV

Targeted proteomics identified tissue inhibitor of metalloproteinase-1 (TIMP-1) as a strong discriminator between active AAV and remission, and between mild and severe disease [101].

Comprehensive cytokine profiling revealed that IL-8, IL-15, and IL-18 binding proteins, nerve growth factor (NGF)-β, thymic and activating regulatory chemokine (TARC), osteopontin, soluble ICAM-1, and KIM-1 distinguish patients more effectively when grouped by ANCA serotype than by clinical subtype, supporting the relevance of ANCA-specific immunopathologic signatures [102].

PTX3 is highly promising: serum and urine PTX3 are elevated in active AAV, correlate with BVAS, and outperform CRP for detecting renal involvement [103]. Anti-PTX3 autoantibodies may help identify ANCA-negative AAV [104].

HMGB1 and calgranulins (S100A8/A9, S100A12) are elevated in AAV and reflect damage-associated molecular pattern (DAMP)-driven inflammation. HMGB1 is particularly associated with renal involvement and granulomatous burden, though findings vary among studies [105,106,107,108,109,110]. S100A8/A9 and S100A12 are elevated in MPO-AAV and predict relapse in PR3-AAV patients treated with rituximab [111,112,113].

Low soluble RAGE (sRAGE) levels support enhanced DAMP-mediated inflammation [110].

### 4.8. Emerging Urinary and Renal Biomarkers in AAV

Urinary PTX3 correlates with active renal vasculitis and reflects albuminuria and estimated glomerular filtration rate (eGFR) decline [103]. Urinary HMGB1 correlates with ESR, CRP, and BVAS and indicates intrarenal inflammation [114].

Urinary KIM-1 and other renal injury molecules have shown discriminatory potential, particularly across ANCA serotypes, though validation remains limited [102].

### 4.9. Clinical Utility and Limitations in AAV

AAV is associated with numerous circulating and urinary biomarkers reflecting inflammation, neutrophil activation, complement consumption, endothelial injury, and DAMP-mediated pathways. Traditional inflammatory markers remain widely used but lack specificity, and ANCA titers, while essential for diagnosis and classification, do not reliably predict relapse across all patients. Low C3 at diagnosis consistently predicts worse renal outcomes.

Among emerging biomarkers, PTX-3 is particularly promising due to its vascular-wall origin, correlation with disease activity, and reduced susceptibility to glucocorticoid suppression. HMGB1 and S100 proteins reflect DAMP-mediated inflammation and may aid relapse prediction. TIMP-1 discriminates active from quiescent disease and stratifies severity. Urinary biomarkers, including HMGB1, PTX3, and KIM-1, reflect intrarenal inflammation and may improve assessment of renal involvement, although larger studies are required. Overall, most biomarkers remain investigational and require robust validation before integration into routine practice.

### 4.10. Cryoglobulinemic Vasculitis

Biomarker studies in cryoglobulinemic vasculitis (CV) are scarce. IL-6 is higher in active CV than in remission [115]. Interferon-related chemokines, particularly C-X-C motif chemokine ligand 10 (CXCL10/IP-10), are elevated in hepatitis C-associated mixed cryoglobulinemia [116,117]. However, their diagnostic and prognostic utility remains unproven.

### 4.11. Hypocomplementemic Urticarial Vasculitis

Validated biomarkers are essentially absent. Persistent hypocomplementemia, particularly low C1q, C3, and C4, is the hallmark laboratory abnormality and part of the diagnostic criteria. Anti-C1q antibodies support diagnosis but do not track activity. No cytokine, chemokine, or endothelial marker has demonstrated reproducible clinical utility [1].

## 5. Biomarkers in Variable-Vessel Vasculitis

Variable-vessel vasculitis comprises disorders affecting arteries, veins, and capillaries of multiple sizes. Behçet’s disease (BD) and Cogan’s syndrome are the principal conditions in this category, although biomarker research is almost entirely concentrated on BD. Cogan’s syndrome remains limited to anecdotal reports and lacks validated biomarkers [118].

### 5.1. Current Clinical and Genetic Markers in BD

BD demonstrates a strong association with HLA-B51, which contributes to genetic susceptibility and varies across phenotypes, with higher prevalence in males and in patients presenting with ocular, genital, and cutaneous disease [119,120]. Nevertheless, HLA-B51 lacks diagnostic specificity and is not included in classification criteria [121].

Routine inflammatory markers such as ESR and CRP often increase during active BD but lack specificity [122,123]. Hematologic indices, especially increased NLR and reduced MPV, correlate with disease severity and vascular involvement [124,125,126,127]. Higher NLR has been observed in neuro-Behçet and in patients with active genital ulcers [127]. Composite inflammation–nutrition indices, such as the CAR, also reflect systemic inflammatory burden but remain nonspecific [120].

### 5.2. Serum Inflammatory Biomarkers and Cytokines in BD

SAA is frequently elevated in BD and is associated with oral ulcers, neurologic involvement, and ocular disease. Persistently high SAA levels may correlate with thrombotic risk and ocular relapse [128,129,130]. SAA also shows an association with IgD levels in mucocutaneous BD [131].

Markers of endothelial dysfunction, including vWF, soluble ICAM-1, VCAM-1, and endothelial microparticles, are increased particularly in vasculo-BD, although their diagnostic discrimination is limited [132,133]. Homocysteine is often elevated in BD and may contribute to thrombosis risk.

Numerous cytokines show consistent elevation in BD, including TNF-α, IL-1β, IL-2, IL-6, IL-8, IL-17, IL-18, and IL-23 [134,135,136,137,138]. IL-6 correlates with arthritic symptoms and major organ inflammatory events [135]. IL-18 increases during ocular flares [137]. IL-23 is elevated in uveitis [139]. IL-8 is strongly associated with mucocutaneous and vascular involvement and may be more sensitive than ESR or CRP for detecting active disease [135,136].

Salivary cytokines mirror mucosal inflammation. IL-1β, IL-8, TNF-α, and IL-6 are increased in BD patients with oral ulcers and vary with disease activity, supporting potential for noninvasive monitoring [134].

Alarmins such as HMGB1 and S100 proteins (S100A8/A9, S100A12) are elevated in BD, but correlations with disease activity are inconsistent, limiting their value as clinical biomarkers [140,141,142,143,144].

Circulating microRNAs may differentiate BD from controls and reflect thrombo-inflammatory pathways, but their use is still investigational.

### 5.3. Urinary Metabolomic and Proteomic Biomarkers in BD

Urine-based metabolomics has identified panels of discriminatory metabolites separating BD patients from healthy controls, although findings require external validation [145]. Urinary proteomic profiling in BD-associated uveitis has identified differentially expressed proteins such as CD38, dipeptidyl peptidase-4 (DPP4), creatine kinase B-type, and S100A8/A9, suggesting potential relevance for monitoring ocular inflammation [146].

### 5.4. Clinical Utility and Limitations of Biomarkers in BD

Despite extensive investigation, no biomarker has demonstrated adequate sensitivity, specificity, or reproducibility for routine diagnostic or monitoring use in BD. HLA-B51 reflects genetic susceptibility but lacks diagnostic power. ESR, CRP, NLR, and MPV provide general inflammatory assessment but are nonspecific. Serum amyloid A and cytokines such as IL-6, IL-8, IL-18, and IL-23 show consistent associations with disease activity and specific organ involvement, yet standardized thresholds and multicenter validation are lacking.

Markers of endothelial dysfunction and alarmins such as HMGB1 and S100 proteins offer mechanistic relevance but inconsistent clinical utility. MicroRNAs and proteomic or metabolomic urinary signatures remain exploratory. Overall, biomarker development in BD remains limited by heterogeneous study designs and small cohorts.

## 6. Single-Organ Vasculitis

Primary angiitis of the central nervous system (PACNS) is a rare vasculitis restricted to the brain and spinal cord. It presents with acute or subacute neurologic or psychiatric symptoms that frequently resemble alternative neurologic disorders, complicating diagnosis [147,148,149]. Biomarker research in PACNS remains limited, especially in children. The only pediatric study to date reported increased levels of vWF antigen in 39 children with childhood-onset PACNS, with levels declining in parallel with clinical improvement. vWF correlated more closely with disease activity than ESR or CRP, suggesting value as a marker of vascular inflammation in this population [150].

In adults, additional biomarker candidates have been proposed. Soluble triggering receptor expressed on myeloid cells 2 (sTREM2) is elevated in both serum and cerebrospinal fluid (CSF), particularly in patients with poorer outcomes. sTREM2 levels correlate with lesion volume and inflammatory mediators such as TNF-α, IL-8, IL-6, and complement components, indicating involvement of myeloid activation in PACNS pathobiology [151].

CECs, reflecting endothelial injury, are increased in active PACNS compared with remission, healthy controls, and non-inflammatory vasculopathies, including reversible cerebral vasoconstriction syndrome and moyamoya disease. These findings suggest that CEC may aid both differential diagnosis and assessment of ongoing inflammatory activity [152].

Persistently elevated CSF IL-17 production has been demonstrated in patients with PACNS compared with non-inflammatory neurological controls. In stroke presentations, increased IL-17 production distinguished PACNS with high specificity, supporting its potential use in select diagnostic contexts [153].

A mass spectrometry-based CSF proteomic study identified 14 proteins, including the amyloid-β A4 protein, present at lower concentrations in PACNS than in controls. These findings suggest neuroaxonal injury and support the potential of CSF proteomics for the discovery of new biomarkers, though all candidates require validation in larger cohorts [154].

## 7. Perspectives on Novel Biomarker Discovery in Vasculitis

While numerous inflammatory biomarkers have been described in vasculitis, recent research increasingly focuses on identifying biomarkers that reflect underlying molecular and genetic mechanisms of disease. Genetic studies have provided important insights into disease susceptibility and heterogeneity across vasculitis subtypes. Genome-wide association studies have identified associations between specific HLA alleles and immune-regulatory genes with diseases such as GCA, TA, AAV, and BD, supporting a role for genetically driven immune dysregulation and offering a framework for genetically informed biomarker discovery [119,120,155,156,157].

Molecular profiling approaches have further expanded biomarker research beyond traditional serum inflammatory markers. Transcriptomic analyses of blood and tissue samples have identified disease- and activity-associated gene expression signatures, particularly involving interferon pathways, Th1/Th17 polarization, neutrophil activation, and complement-related genes in AAV and LVV [28,97,98,101,102,111,112].

Proteomic and metabolomic studies have enabled the identification of circulating and urinary biomarkers linked to endothelial injury, extracellular matrix remodeling, and organ-specific damage, such as urinary biomarkers reflecting intrarenal inflammation in IgAV and AAV [74,83,87,101,114,145,146].

**Figure 4:**
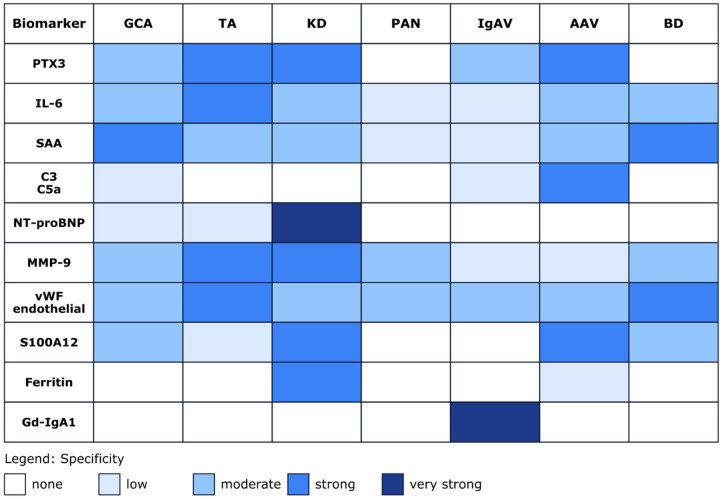
Biomarker specificity heatmap across vasculitis types. The heatmap illustrates the relative association and reported specificity of selected biomarkers across different vasculitis subtypes. The color intensity reflects the strength and consistency of associations reported in the literature. Color gradients indicate relative trends reported across studies and should not be interpreted as quantitative effect sizes. The heatmap was compiled by the authors based on a qualitative synthesis of published studies and expert interpretation of the available evidence, rather than derived from a single dataset or quantitative meta-analysis. Where evidence was inconsistent across studies, we assigned a lower intensity to reflect uncertainty. The selected references for individual biomarkers are as follows. PTX3: [20,21,22,78,103,158,159,160]; IL-6: [17,23,24,26,27,38,59,60,161,162,163]; SAA: [13,18,59,128,129,130,164,165]; C3, C5a: [83,98,166]; NT-proBNP: [37,167]; MMP-9: [23,45,56,168,169]; vWF/endothelial markers: [99,170,171,172,173]; S100A12: [34,46,47,111,112,113,144]; Ferritin: [49,174]; and Gd-IgA1: [72,73,74,75]. The list of abbreviations is as follows: AAV: ANCA-associated vasculitis; BD: Behçet’s disease; C3: complement component 3; C5a: complement component C5a; GCA: giant cell arteritis; Gd-IgA1: galactose-deficient immunoglobulin A1; IgAV: IgA vasculitis; IL-6: interleukin-6; KD: Kawasaki disease; MMP-9: Matrix metalloproteinase-9; NT-proBNP: N-terminal pro–B-type natriuretic peptide; PAN: polyarteritis nodosa; PTX3: pentraxin-3; S100A12: S100 calcium-binding protein A12; SAA: serum amyloid A; TA: Takayasu arteritis; vWF: von Willebrand factor.

Importantly, several of the most promising emerging biomarkers discussed in this review (Figure 4), including PTX3, S100 proteins, complement components, Gd-IgA1, and urinary kidney injury markers, have been identified through mechanistic or molecularly guided approaches rather than unbiased screening alone [20,22,33,66,71,74,87,114]. Future biomarker discovery is therefore likely to benefit from integrative strategies combining genetic susceptibility data, molecular pathway analysis, and longitudinal clinical phenotyping, with the goal of identifying biomarkers that improve disease stratification, activity assessment, and prediction of organ involvement.

## 8. Conclusions

Across vasculitides, biomarker discovery is hindered by small cohort sizes, treatment heterogeneity, lack of standardized outcome measures, and variability in assay methodology. Most candidate biomarkers lack external validation, disease specificity, or incremental predictive performance compared with established clinical and imaging tools. Future progress will require large, prospective, multi-omic studies with harmonized sample processing, integration of vascular imaging endpoints, and evaluation of biomarker panels rather than single analytes.

## Figures and Tables

**Table 1:** Biomarker landscape across vasculitis types.

Vasculitis Group	Representative Diseases	Validated Biomarkers	Emerging Biomarkers
Large Vessel	GCA	ESR, CRP, SAA	PTX3, MMP-9, IL-6
	Takayasu arteritis	ESR, CRP	PTX3, IL-6, MMP-9, VEGF
Medium Vessel	Kawasaki disease	CRP, ESR	IL-6, IL-17, NT-proBNP, MMP-9
	Polyarteritis nodosa	CRP, ESR	HMGB1, MMP-9
Small Vessel	IgA vasculitis	CRP, ESR	Gd-IgA1, AGT, KIM-1, NGAL
	ANCA-associated vasculitis	ANCA, CRP, ESR	C3/C5a, TIMP-1, PTX3
Variable Vessel	Behçet’s disease	CRP, ESR, SAA	IL-6, IL-8, S100A12, ferritin
	Cogan’s syndrome	(none validated)	sTREM2, ferritin

List of abbreviations: AGT: angiotensinogen; ANCA: antineutrophil cytoplasmic antibodies; C3: complement component 3; C5a: complement component C5a; CRP: C-reactive protein; ESR: erythrocyte sedimentation rate; Gd-IgA1: galactose-deficient immunoglobulin A1; HMGB1: high-mobility group box-1 protein; IL-6/IL-8/IL-17: interleukin-6/interleukin-8/interleukin-17; KIM-1: kidney injury molecule-1; MMP-9: Matrix metalloproteinase-9; NGAL: neutrophil gelatinase-associated lipocalin; NT-proBNP: N-terminal pro–B-type natriuretic peptide; PTX3: pentraxin-3; S100A12: S100 calcium-binding protein A12; SAA: serum amyloid A; sTREM2: soluble triggering receptor expressed on myeloid cells-2; TIMP-1: tissue inhibitor of metalloproteinases-1; VEGF: vascular endothelial growth factor.

**Table 2:** Biomarkers by disease and clinical use.

Vasculitis	Diagnosis/Classification	Activity/Monitoring	Prognosis/Complications	Organ-Specific/Other
GCA (LVV)	ESR, CRP, SAA; PTX3	ESR, CRP, SAA; PTX3; IL-6; IL-17/IL-23 axis; MCP-1; MMP-9; S100A8/A9, S100A12	Baseline ESR/CRP/SAA; PTX3; VEGF	MMP-9; VEGF
Takayasu arteritis (LVV)	ESR, CRP; PTX3	ESR, CRP; PTX3; IL-6; IL-17/IL-23 axis; MCP-1; IL-18; VEGF; EPCs; CECs	PTX3; MMP-9; VEGF	VEGF; EPCs; CECs; leptin/adiponectin
Kawasaki disease (MVV)	CRP, ESR; CBC changes (neutrophils, platelets, NLR, MPV); NT-proBNP	CRP, ESR; IL-1β; IL-6; TNF-α; IL-17A/IL-18/IL-23; S100A8/A9 & S100A12; endothelial activation markers (VEGF/adhesion molecules/CECs); MMP-9	Hypoalbuminemia; NT-proBNP; S100A8/A9 & S100A12; MMP-9; galectin-3; ferritin; D-dimer; neutrophil %/NLR	NT-proBNP; MMP-9; endothelial injury panel
Polyarteritis nodosa (MVV)	No validated disease-specific marker; LAMP-2/anti-LAMP-2; anti-PSPT	ESR, CRP; HMGB1; anti-PSPT; anti-moesin; LAMP-2/anti-LAMP-2; sVCAM-1; thrombomodulin; AECA	HMGB1; anti-moesin; LAMP-2/anti-LAMP-2	sVCAM-1; thrombomodulin; AECA; MMP-9
IgA vasculitis (IgAV/IgAVN)	Leukocyte count; CRP; IL-6; SAA; composite indices (SAA, IgA, IgM, CRP)	Th17 cytokines (IL-17A/IL-18/IL-23); TNF-α; calprotectin (S100A8/A9); serum HMGB1	Renal: Gd-IgA1; PTX3; AGT; urinary C3/C4/C5/C5a; urinary HMGB1; urinary KIM-1/MCP-1/NAG/NGAL/e-GST; MMP-9. GI: fecal calprotectin	Renal urinary biomarker panel; fecal calprotectin
AAV	ANCA (MPO, PR3); ESR, CRP	ESR, CRP; ANCA titers; vWF; DNI; TIMP-1; PTX3; HMGB1; S100A8/A9; S100A12; IL-8; IL-15; TARC; osteopontin; sICAM-1; CXCL13; sRAGE	CAR ratio; low C3; S100A8/A9 & S100A12; PTX3	Urinary PTX3; urinary HMGB1; urinary KIM-1
Cryoglobulinemic vasculitis	Cryoglobulins; low C4; HCV markers	IL-6; CXCL10/IP-10	—	Interferon-related chemokines (esp. CXCL10/IP-10)
HUV	Low C1q, C3, C4; anti-C1q	Complement trends (C1q/C3/C4)	—	—
Behçet’s disease	HLA-B51; ESR, CRP; NLR, MPV; CAR	SAA; vWF; IL-1β/IL-6/IL-8/IL-17/IL-18/IL-23; TNF-α; salivary cytokines; sICAM-1/VCAM-1; endothelial microparticles; HMGB1/S100 proteins	SAA; homocysteine; NLR; CAR	microRNAs; urinary metabolomic/proteomic panels
Cogan’s syndrome	anti-Hsp70 (anti-Cogan peptide) antibodies (typical CS); none validated otherwise	ESR, CRP (nonspecific)	—	—
PACNS	CEC; CSF IL-17	vWF antigen; CEC	sTREM2	CSF proteomics

List of abbreviations: AECA: anti-endothelial cell antibodies; AGT: angiotensinogen; ANCA: antineutrophil cytoplasmic antibodies; C1q: complement component 1q; C3: complement component 3; C4: complement component 4; C5: complement component 5; C5a: complement component 5a; CAR: C-reactive protein to albumin ratio; CBC: complete blood count; CECs: circulating endothelial cells; CRP: C-reactive protein; CXCL10/IP-10: C-X-C motif chemokine ligand 10/interferon-γ-induced protein 10; CXCL13: C-X-C motif chemokine ligand 13; D-dimer: fibrin degradation product D-dimer; DNI: delta neutrophil index; e-GST: erythrocyte glutathione S-transferase; EPCs: endothelial progenitor cells; ESR: erythrocyte sedimentation rate; Gd-IgA1: galactose-deficient IgA1; HCV: hepatitis C virus; HLA-B51: human leukocyte antigen B51; HMGB1: high-mobility group box 1; HUV: hypocomplementemic urticarial vasculitis; IL-15: interleukin-15; IL-17: interleukin-17; IL-17A: interleukin-17A; IL-18: interleukin-18; IL-1β: interleukin-1 beta; IL-23: interleukin-23; IL-6: interleukin-6; IL-8: interleukin-8; KIM-1: kidney injury molecule-1; LAMP-2: lysosome-associated membrane protein 2; MCP-1: monocyte chemoattractant protein-1; microRNAs: micro-ribonucleic acids; MMP-9: Matrix metalloproteinase-9; MPO: myeloperoxidase; MPV: mean platelet volume; NAG: N-acetyl-β-D-glucosaminidase; NGAL: neutrophil gelatinase-associated lipocalin; NLR: neutrophil-to-lymphocyte ratio; NT-proBNP: N-terminal pro-B-type natriuretic peptide; PACNS: primary angiitis of the central nervous system; PR3: proteinase-3; PTX3: pentraxin-3; S100A12: S100 calcium-binding protein A12; S100A8/A9: calprotectin; SAA: serum amyloid A; sICAM-1: soluble intercellular adhesion molecule-1; sRAGE: soluble receptor for advanced glycation end products; sTREM2: soluble triggering receptor expressed on myeloid cells 2; sVCAM-1: soluble vascular cell adhesion molecule-1; TARC: thymus and activation-regulated chemokine; TIMP-1: tissue inhibitor of metalloproteinases-1; TNF-α: tumor necrosis factor alpha; VCAM-1: vascular cell adhesion molecule-1; VEGF: vascular endothelial growth factor; vWF: von Willebrand factor.

## Data Availability

No new data were created or analyzed in this study. Data sharing is not applicable to this article.
